# Miller Fisher Variant of Guillain-Barré Syndrome Presenting With Dysphagia and Ophthalmoplegia

**DOI:** 10.7759/cureus.100614

**Published:** 2026-01-02

**Authors:** Rupam Sharma, Sanjana Murdande, Jose Garcia-Corella, Arin Orogian, Linh Tran

**Affiliations:** 1 Internal Medicine, University of California, Los Angeles (UCLA) Kern Medical, Bakersfield, USA

**Keywords:** areflexia, dysphagia, gbs treatment, guillain-barre syndrome (gbs), miller fisher variant, ophthalmoplegia

## Abstract

Miller Fisher syndrome (MFS) is a rare variant of Guillain-Barré syndrome (GBS). It is characterized by a triad of ataxia, areflexia, and ophthalmoparesis. Akin to GBS, it is often triggered by a respiratory infection, causing immune cross-activation against pathogenic proteins and gangliosides. Symptoms typically occur in chronological progression, beginning with ophthalmoparesis, then ataxia, and finally areflexia. Our case is of a patient presenting with dysphagia and ophthalmoplegia as the initial manifestations. There currently exist only a limited number of reported cases of MFS presenting with dysphagia, which, along with ophthalmoplegia, areflexia, and ataxia, should raise suspicion for MFS.

## Introduction

Guillain-Barré syndrome (GBS) is a rare but potentially life-threatening autoimmune disorder characterized by acute peripheral neuropathy, often triggered by an infection. While the classic form of GBS typically presents with symmetric ascending muscle weakness and areflexia, several variants exist, each with distinct clinical features. One such variant is Miller Fisher syndrome (MFS), which is considered a specific form of GBS. It accounts for 1-5% of all GBS cases in Western countries [1]. MFS is characterized by the classical triad of ataxia, areflexia, and ophthalmoplegia, and it is often associated with anti-GQ1b antibodies, which are thought to play a crucial role in the pathogenesis of the disease. The localization of GQ1b antigens to the oculomotor nerve and muscle spindles is responsible for the characteristic features. The glossopharyngeal and vagus nerves may also express the GQ1b antigen, producing dysphagia in some patients [2,3]. However, only 2% of patients have been reported to present with dysphagia as the initial symptom, perhaps because the responsible nerves are less affected, which contributes to the diagnostic challenge [4]. Though MFS is rare, its recognition is important, as early diagnosis and appropriate treatment can significantly improve patient outcomes. Here is presented a unique case of a patient with the Miller Fisher variant of GBS presenting with severe dysphagia, which initially was thought to be myasthenia gravis.

## Case presentation

The patient was a 35-year-old White male with a medical history of syphilis one year ago treated with penicillin at a public county hospital and gastric sleeve 10 years ago with resulting 100 pound weight loss, who presented to the emergency department (ED) with chief complaint of dysphonia, blurry vision, weakness and numbness in arms and legs, and difficulty swallowing solids and liquids, which began suddenly one day prior to presentation. Upon presentation, stroke code was activated, and the patient underwent computed tomography (CT), magnetic resonance imaging (MRI), and magnetic resonance angiography (MRA) of the head, all of which resulted in negative findings. The respiratory BioFire test was negative as well. The patient was transferred to another hospital for higher-level care for neurology services, where the initial working diagnosis was myasthenia gravis (MG), and the patient received three doses of pyridostigmine. The patient noted minor improvement in his blurry vision and ptosis, following which the plan was to initiate plasmapheresis, which was unavailable at that hospital. Therefore, the patient was transferred to our hospital for plasmapheresis.

Upon arrival at our facility, initial vital signs were significant for blood pressure of 155/79 mmHg, heart rate of 64 beats per minute, respiratory rate of 17 breaths per minute, and >98% oxygen saturation on room air. On physical exam, the patient was a well-developed young male with a nasogastric tube and severe dysarthria and dysphonia. He had a weak cough and had to use suction at bedside due to his inability to swallow his saliva. He also needed to pinch his nose to clearly speak. He had a lateral gaze palsy, bilateral superior gaze palsy, and right-sided ptosis. Neck flexion strength was three out of five, head extension was five out of five, while muscle strength in the deltoids, finger extensors, opponens pollicis, dorsal interossei, and psoas and hamstring muscles was four out of five. Triceps and biceps were five out of five. Reflexes were absent. He had decreased sensation to pinprick distally to mid-thighs and elbows, and a moderate decrease in position sensation of toes and fingers. He had severe ataxia and was unable to walk in a straight line. Initial labs revealed thyroid-stimulating hormone of 1.4 (reference range: 0.4 to 4 μU/mL), B12 of 914 (reference range: 193 to 986 pg/mL), and folate of 9.1 (reference range: >3.1 ng/mL). Urine toxicology was unremarkable. Speech language pathology services evaluated the patient due to dysphagia and noted decreased labial seal and reduced hyolaryngeal elevation.

Neurology was consulted, and the patient was initiated on intravenous immunoglobulin (IVIG) 0.5 g/kg daily, beginning the day after onset of illness, for four days total. While the standard IVIG treatment for GBS is 0.4 g/kg/day for five days, this regimen provided the same quantity of IVIG over a shorter duration. The MG antibody panel was negative for acetylcholine receptor blocking, binding, and modulating antibodies, as well as anti-muscle-specific kinase antibodies. Anti-LRP4 antibody was not tested as it has a sensitivity of 1-50% in patients with MG, and there is inconclusive evidence on its specificity and utility for diagnosis [5]. Lumbar puncture revealed cerebrospinal fluid (CSF) with white blood cell count of 1 (reference range: <5 cells), zero red blood cells (reference range: <1 cell), glucose of 69 mg/dL (reference range: 40 to 75 mg/dL), and protein of 35.7 mg/dL (reference range: 15 to 45 mg/dL). Electromyography and nerve conduction studies were done three days after the onset of illness (Tables 1-4). Nerve conduction study of the right upper and lower limbs demonstrated severe diffuse sensory neuropathy with absent median, ulnar, and superficial peroneal and sural sensory potentials, as well as absent right median and ulnar F waves, which can be seen with GBS. Electromyography of the right upper and lower limbs showed mild chronic denervation in the right extensor digitorum brevis, tibialis anterior, and vastus medialis due to a chronic neuropathy. Anti-GQ1b antibody, which resulted in two weeks, was elevated at a 1:6400 titer (reference range: <1:100 titer). No other antibodies were tested.

**Table 1:** Electromyography summary. N: normal; SI Inc: signal intensity increased.

Muscle	Nerve	Roots	Insertional activity	Fibrillations	Positive sharp waves	Fasciculations	Amplitude	Polyphasic	Duration	Recruitment pattern
Right deltoid	Axillary	C5-C6	N	None	None	None	N	None	N	N
Right biceps brachii	Musculocutaneous	C5-C6	N	None	None	None	N	None	N	N
Right triceps brachii	Radial	C6-C8	N	None	None	None	N	None	N	N
Right extensor digitorum communis	Radial	C7-C8	N	None	None	None	N	None	N	N
Right first dorsal interosseous	Ulnar	C8-T1	N	None	None	None	N	None	N	N
Right tibialis anterior	Deep peroneal (fibular)	L4-L5	N	None	None	None	SI Inc	None	N	N
Right gastrocnemius	Tibial	S1-S2	N	None	None	None	N	None	N	N
Right vastus medialis	Femoral	L2-L4	N	None	None	None	SI Inc	None	N	N
Right rectus femoris	Femoral	L2-L4	N	None	None	None	N	None	N	N
Right extensor digitorum brevis	Tibial	L5-S1	N	None	None	None	SI Inc	None	N	N

**Table 2:** Motor nerve conduction study. Ref.: reference; APB: abductor pollicis brevis; ADM: abductor digiti minimi; EDB: extensor digitorum brevis; AH: abductor hallucis.

Nerve/sites	Muscle	Latency (milliseconds)	Amplitude (millivolts)	Duration (milliseconds)	Segments	Distance (centimeters)	Latency difference (milliseconds)	Velocity (meters/second)	Temperature (degrees Celsius)
Right median - abductor pollicis brevis (APB)									
Wrist	APB	3.18	12	7.24	Wrist - APB	7			31.6
Ref.		≤4.4	≥4.5		Ref.				
Elbow	APB	6.93	11.2	7.45	Elbow - wrist	23	3.75	61	32.9
Ref.					Ref.			≥49	
Right ulnar - abductor digiti minimi (ADM)									
Wrist	ADM	2.34	10	6.72	Wrist - ADM	7			33.9
Ref.		≤4.20	≥4.5		Ref.				
Below elbow	ADM	5.73	10.9	6.77	Below elbow - wrist	22	3.39	65	33.9
Ref.					Ref.			≥49	
Above elbow	ADM	6.88	10.5	6.72	Above elbow - Below elbow	10	1.15	87	34.4
Ref.					Ref.			≥49	
					Above elbow - wrist		4.53		34.4
Right peroneal - extensor digitorum brevis (EDB)									
Ankle	EDB	4.17	5.1	8.54	Ankle - EDB	9			33.5
Ref.		≤6.20	≥2.4		Ref.				
Fibular head	EDB	9.53	4.7	8.96	Fibular head - ankle	26	5.36	48	33.6
Ref.					Ref.			≥39	
Popliteal fossa	EDB	11.35	4.7	7.6	Popliteal fossa - fibular head	10	1.82	55	33.5
Ref.					Ref.			≥39	
					Popliteal fossa - ankle		7.19		33.5
Right tibial - abductor hallucis (AH)									
Ankle	AH	3.49	8.7	6.51	Ankle - AH	8			33.8
Ref.		≤6	≥5		Ref.				
Popliteal fossa	AH	10.99	7.9	7.24	Popliteal fossa - ankle	42	7.5	56	33.8
Ref.					Ref.			≥40	

**Table 3:** Sensory nerve conduction study. Ref.: reference; Dig: digit; NR: no response.

Nerve/sites	Recording site	Peak latency (milliseconds)	Amplitude (microvolts)	Segments	Distance (centimeters)	Temperature (degrees Celsius)
Right median - digit II (antidromic)						
Wrist	Dig II	NR	NR	Wrist - Dig II	14	33.5
Ref.		≤3.80	≥10	Ref.		
Right ulnar - digit V (antidromic)						
Wrist	Dig V	NR	NR	Wrist - Dig V	14	33.4
Ref.		≤3.80	≥9	Ref.		
Right median, radial - thumb comparison						
Median wrist	Thumb	NR	NR	Median wrist - thumb	10	34.3
Ref.		≤3	≥9	Ref.		
Radial wrist	Thumb	NR	NR	Radial wrist - thumb	10	34
Ref.		≤3	≥4	Ref.		
				Median wrist - radial wrist		34
Right superficial peroneal - ankle						
Lateral leg	Ankle	NR	NR	Lateral leg - ankle	14	33.9
Ref.		≤4.40	≥4	Ref.		
Right sural - ankle (calf)						
Calf	Ankle	NR	NR	Calf - ankle	14	33.9
Ref.		≤4.40	≥4	Ref.		

**Table 4:** F wave study. NR: no response; F: F wave; M: motor; APB: abductor pollicis brevis; ADM: abductor digiti minimi; EDB: extensor digitorum brevis; AH: abductor hallucis.

Nerve	F latency (milliseconds)	Reference (milliseconds)	M latency (milliseconds)	F-M latency (milliseconds)
Right median - APB	NR	≤32	3.2	3.1
Right ulnar - ADM	NR	≤32	2.8	2.8
Right peroneal - EDB	50.3	≤60	4.3	45.9
Right tibial - AH	50.7	≤60	3.9	46.8

The patient significantly improved following IVIG therapy. At the three-month neurology clinic visit, he no longer had difficulty swallowing, could run up to 8 miles, and could lift up to 100 lbs. in the gym. He continued to have mild weakness with mastication, intermittent double vision only when looking to the left, and intermittent numbness in arms and legs during awake periods, two to three times per week. He had five out of five strength in bilateral upper and lower extremities with normal sensation to pinprick; however, he continued to have mild weakness in left eye abduction. Reflexes were absent, except for hyporeflexic patellar reflexes.

## Discussion

GBS exists as a spectrum with variants including MFS. MFS, also known as anti-GQ1b antibody syndrome, is considered a variant of GBS due to its clinical and pathophysiological similarities as an acute immune-mediated polyneuropathy. Classic MFS is characterized by the triad of ataxia, areflexia, and ophthalmoplegia, which follows the sequential pattern of clinical appearance, worsening, and recovery seen in GBS [3]. Non-classic or incomplete MFS has been described with less frequency, with a limited number of case reports and case series reporting a wide spectrum of symptoms, such as ptosis, mydriasis, pharyngeal-cervical-brachial weakness, ataxic neuropathy, oropharyngeal palsy, and Bickerstaff brainstem encephalitis [6,7]. The pharyngeal-cervical-brachial variant of GBS, in particular, presents with oropharyngeal and cervicobrachial weakness as well as upper extremity areflexia. Lower extremity strength is usually preserved, unlike in our patient, who presented with ataxia, lower extremity weakness, and areflexia [8]. It is possible that our patient had an overlap of seronegative MG with MFS. Seronegative MG is defined as MG without detectable auto-antibodies, most commonly the anti-acetylcholine receptor antibodies and the anti-muscle-specific kinase antibodies [9]. There exist five cases in the literature of MG and MFS overlap. One of these cases was that of a patient diagnosed with seronegative MG 15 years prior to MFS diagnosis [10]. Our patient had never experienced symptoms characteristic of MG prior to hospitalization. His presenting examination with diplopia and ptosis was concerning for MG. While he had a slight improvement in his symptoms with pyridostigmine, the MG antibody panel tested negative. While tests of fatiguability were not conducted for more specific characterization of ocular symptoms, he had significant improvement in eye motility over time without the use of acetylcholinesterase inhibitors, steroids, plasmapheresis, or other standard therapies for MG. Instead, his symptoms improved following IVIG therapy, more consistent with MFS.

It is hypothesized that MFS spawns as an autoimmune response to a recent infection, most commonly *Campylobacter jejuni*, cytomegalovirus, and Epstein-Barr virus [11], with a small number of cases seen following COVID-19 vaccination [12]. Although the exact mechanism is not well understood, interaction between peripheral nerve and infectious antigens is thought to trigger the adaptive immune system and development of antibodies against gangliosides, most frequently IgG anti-GQ1b (~85%) [2-7]. Seronegative MFS has shown a variety of antibodies against other gangliosides [13]. Having a similar pathogenesis, it is undetermined why some patients develop GBS while others present with MFS. The diagnosis of MFS is based on clinical examination and is supported by laboratory and electrophysiologic testing, such as lumbar puncture, electromyography, and nerve conduction studies, as well as anti-GQ1b antibody titer, all of which were characteristic of MFS in this patient [14]. Lumbar puncture typically shows albuminocytologic dissociation, although if absent, it does not rule out MFS as a diagnosis. Electrodiagnostic studies can show reduced sensory responses, and finally, antibody testing is important to support the diagnosis.

## Conclusions

The initial presentation was challenging due to severe dysphagia and dysphonia in conjunction with ophthalmoplegia and arm and leg weakness and numbness. Differentiation between oropharyngeal and esophageal dysphagia by evaluating the swallow function was key to determining the presence of bulbar muscle weakness. These symptoms, along with unilateral ptosis and blurry vision, initially raised suspicion for MG. It is possible that the patient had an overlap of seronegative MG and MFS, and tests of fatiguability and the neostigmine test can be considered for further delineation in future similar patients. Ultimately, the positive IgG anti-GQ1b antibodies supported the diagnosis of MFS, by which time the patient had significantly improved due to the use of IVIG. In summary, despite the classically described triad of ataxia, areflexia, and ophthalmoplegia, MFS may present with a variety of symptoms, including bulbar palsy causing severe dysphagia and dysphonia.
